# Mechanosensing by the Primary Cilium: Deletion of Kif3A Reduces Bone Formation Due to Loading

**DOI:** 10.1371/journal.pone.0033368

**Published:** 2012-03-12

**Authors:** Sara Temiyasathit, W. Joyce Tang, Philipp Leucht, Charles T. Anderson, Stefanie D. Monica, Alesha B. Castillo, Jill A. Helms, Tim Stearns, Christopher R. Jacobs

**Affiliations:** 1 Bone and Joint Rehabilitation R&D Center, Department of Veterans Affairs, Palo Alto, California, United States of America; 2 Department of Bioengineering, Stanford University, Stanford, California, United States of America; 3 Department of Biological Sciences, Stanford University, Stanford, California, United States of America; 4 Department of Biomedical Engineering, Columbia University, New York, New York, United States of America; 5 Department of Genetics, Stanford University, Stanford, California, United States of America; 6 Department of Mechanical Engineering, Stanford University, Stanford, California, United States of America; 7 Department of Orthopaedic Surgery, Stanford University, Stanford, California, United States of America; 8 Department of Surgery-Plastic and Reconstructive Surgery, Stanford University, Stanford, California, United States of America; 9 Energy Biosciences Institute, University of California, Berkeley, California, United States of America; The University of Akron, United States of America

## Abstract

Primary cilia, solitary microtubule-based structures that grow from the centriole and extend into the extracellular space, have increasingly been implicated as sensors of a variety of biochemical and biophysical signals. Mutations in primary cilium-related genes have been linked to a number of rare developmental disorders as well as dysregulation of cell proliferation. We propose that primary cilia are also important in mechanically regulated bone formation in adults and that their malfunction could play a role in complex multi-factorial bone diseases, such as osteoporosis. In this study, we generated mice with an osteoblast- and osteocyte-specific knockout of Kif3a, a subunit of the kinesin II intraflagellar transport (IFT) protein; IFT is required for primary cilia formation, maintenance, and function. These *Colα1(I) 2.3-Cre;Kif3a^fl/fl^* mice exhibited no obvious morphological skeletal abnormalities. Skeletally mature *Colα1(I) 2.3-Cre;Kif3a^fl/fl^* and control mice were exposed to 3 consecutive days of cyclic axial ulna loading, which resulted in a significant increase in bone formation in both the conditional knockouts and controls. However, *Colα1(I) 2.3-Cre;Kif3a^fl/fl^* mice did exhibit decreased formation of new bone in response to mechanical ulnar loading compared to control mice. These results suggest that primary cilia act as cellular mechanosensors in bone and that their function may be critical for the regulation of bone physiology due to mechanical loading in adults.

## Introduction

Primary cilia are nearly ubiquitous microtubule-based cellular structures that, although discovered almost a century ago, have received less attention than the motile cilia found in the respiratory and reproductive tracts. However, a recent flurry of research into the function of the non-motile primary cilium has implicated it in the Sonic hedgehog (Shh) [1], [2], [3], [4], Wnt [5], [6], and platelet derived growth factor (PDGF) [7] signaling pathways, as well as mechanosensing in the kidney [8], [9], [10] and embryonic node [11]. The emerging picture is that the primary cilium acts as a versatile nexus where extracellular signals are sensed and integrated to initiate a coordinated cellular response. In terms of human disease, mutations in primary cilium proteins have been linked to a number of rare developmental disorders as well as the dysregulation of cell proliferation [12]. However, the role of primary cilia in complex multi-factorial bone diseases, such as osteoporosis, is unknown.

A number of important tissues and organs are regulated not only by biochemical but also by mechanical factors. Abnormal response to mechanical loading leads to the development of diseases such as osteoporosis and atherosclerosis [13]. Understanding the mechanism by which the cells within these tissues sense mechanical loads is critical in understanding the etiology of such diseases. Primary cilia have been shown to act as mechanosensors in the kidney and liver [8], [9], [14], regulating cell proliferation in these tissue types. There is *in vitro* evidence that they act as mechanosensors in bone as well, with cultured osteoblasts and osteocytes responding to mechanical stimulation with primary cilium-dependent osteogenic gene expression [15]. Germ line deletion of the primary cilium-associated gene Pkd1 in mice results in polycystic kidney disease and reduced bone mass [16], however this finding is difficult to interpret due to potential cross-talk between bone and impaired kidney function. Conditional deletion of Pkd1 in bone tissue results in skeletal developmental abnormalities [17], with reduced bone formation in development of the cranium that has been ascribed to abnormal sensing of mechanical loads during cranial expansion [18]. It is unclear how these findings relate to adult human bone pathologies such as osteoporosis because the cranial stresses and strains that occur during development are difficult to relate to the skeletal loading in adults that is due to habitual activities such as ambulation.

In this study, we investigate the role of primary cilia in regulating skeletal mechanosensing *in vivo*. Since genetic ablation of the primary cilium is embryonic lethal, we used mice with a conditional deletion of Kif3a, an essential subunit of the kinesin II intraflagellar transport (IFT) motor protein, in osteoblasts and osteocytes. Intraflagellar transport along the microtubule core of the primary cilium is required for its formation, maintenance, and function [19], [20]. Deletion of Kif3a has been linked to cell signaling dysfunction through disruption of IFT [4], [21]. Using this method of primary cilium disruption, we found no substantial difference in skeletal morphology in embryonic and skeletally mature mice without loading, suggesting that primary cilia in osteoblasts and osteocytes do not play a role in skeletal development. We show, however, that a tissue-specific conditional knockout of Kif3a in mice leads to attenuated sensitivity of bone to mechanical loading. This suggests that primary cilia are important for the regulation of bone formation in adults and may be involved in a larger number of complex human diseases than previously appreciated.

## Results

**Figure 1:**
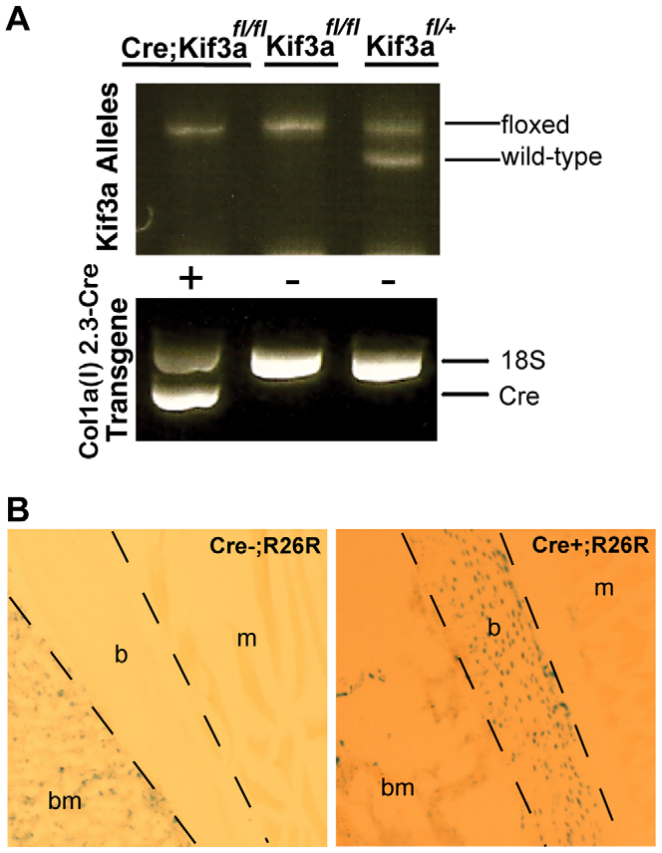
Generation and confirmation of bone-specific Kif3a conditional knockout mice. (**A**) A typical agarose gel resulting from PCR genotyping of genomic DNA from tail biopsies of transgenic mice. Bands indicate floxed (490 bp) and wild-type (360 bp) *Kif3a* and *Cre* recombinase (650 bp). 18S (870 bp) used as a positive control in the *Cre* PCR reactions. Floxed and recombined *Kif3a* allele present in *Colα1(I) 2.3-Cre;Kif3a^fl/fl^* mice due to heterogeneous tissue in tail biopsies. (**B**) To assess *Cre* specificity, *Colα1(I) 2.3-Cre* mice were crossed with *Rosa26R* reporter mice. Effective *Cre* recombination was detected by LacZ staining in osteoblasts and osteocytes of *Colα1(I) 2.3-Cre;R26R* mice (right) but not in littermates lacking *Cre* (left). LacZ staining was not visible in muscle tissue of *Colα1(I) 2.3-Cre;R26R* mice. (b- bone, bm- bone marrow, m- muscle).

We first generated mice with a bone-specific knockout of *Kif3a*, a subunit of kinesin II which is required for functional primary cilia, by crossing mice possessing a floxed *Kif3a* allele with mice expressing *Cre* recombinase specifically in bone driven by the 2.3 kb fragment of the α1(I)-collagen promoter [22], [23]. Resulting *Colα1(I) 2.3-Cre;Kif3a^fl/+^* and *Kif3a^fl/+^* offspring were selected for subsequent breeding to generate *Colα1(I) 2.3-Cre;Kif3a^fl/fl^* (experimental) mice and control mice, which were identified by PCR-based genotyping of genomic DNA (Fig. 1A). The growth and survival of *Colα1(I) 2.3-Cre; Kif3a^fl/fl^* mice were similar to that of wild-type controls. To assess the specificity of *Cre* expression, *Colα1(I) 2.3-Cre* mice were crossed with *Rosa26R* reporter mice, and effective Cre recombination was detected by LacZ staining in osteoblasts and osteocytes of *Colα1(I) 2.3-Cre;R26R* mice but not in littermates lacking *Cre* (Fig. 1B). Cre recombination did not occur in non-bone tissue of *Colα1(I) 2.3-Cre* mice.

**Figure 2:**
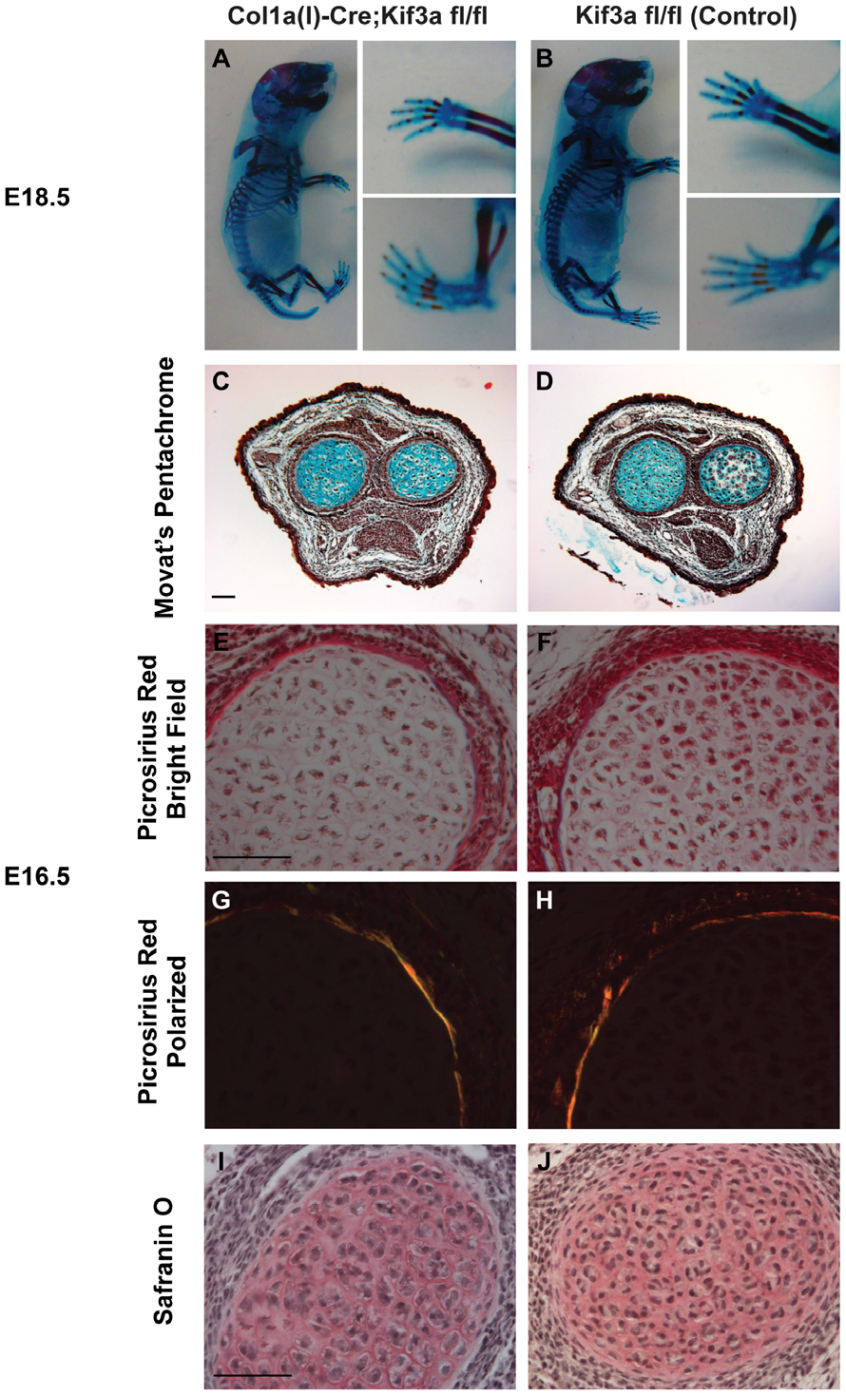
Kif3a expression in osteoblasts and osteocytes is not critical for embryonic skeletal development. (**A,B**) Whole mount Alizarin Red (bone) and Alcian Blue (cartilage) staining of E18.5 *Colα1(I) 2.3-Cre;Kif3a^fl/f^* ^l^ (A) and control (B) embryos. The size and limb patterning of *Colα1(I) 2.3-Cre;Kif3a^fl/fl^* mice was similar to that of the control mice. (**C,D**) Movat's pentachrome staining of cross-sections of the radial/ulnar growth plates (cartilage-blue) in E16.5 *Colα1(I) 2.3-Cre;Kif3a^fl/f^* ^l^ (C) and control (D) mice. (**E–J**) Cross-sections of E16.5 long bones stained with Picrosirius red (E,F-bright field; G,H-polarized light) to illuminate collagen and Safranin O (I,J) to demarcate cartilage. Both control and *Colα1(I) 2.3-Cre;Kif3a^fl/f^* ^l^ mice have similar patterns of osteogenic and chondrogenic differentiation. Scale bar: 100 µm.

To determine whether *Colα1(I) 2.3-Cre;Kif3a^fl/fl^* mice exhibit abnormalities in skeletal morphology, we compared embryos at different developmental stages using histological and whole mount techniques. Embryo size and limb patterning were indistinguishable between E18.5 *Colα1(I) 2.3-Cre;Kif3a^fl/fl^* and control mice (Fig. 2A,B). There were also no differences between E16.5 *Colα1(I) 2.3-Cre;Kif3a^fl/fl^* and control mice with regards to osteogenic (Fig. 2C–H) and chondrogenic (Fig. 2C,D,I,J) differentiation. These data indicate that primary cilia in osteoblasts and osteocytes are not critical for embryonic viability, unlike the systemic knockout of *Kif3a*, which is embryonic lethal [24]. Also, disruption of *Kif3a* in osteoblasts and osteocytes does not affect skeletal size and patterning, unlike *Kif3a* deletion in messenchyme tissue [25], [26].

**Figure 3:**
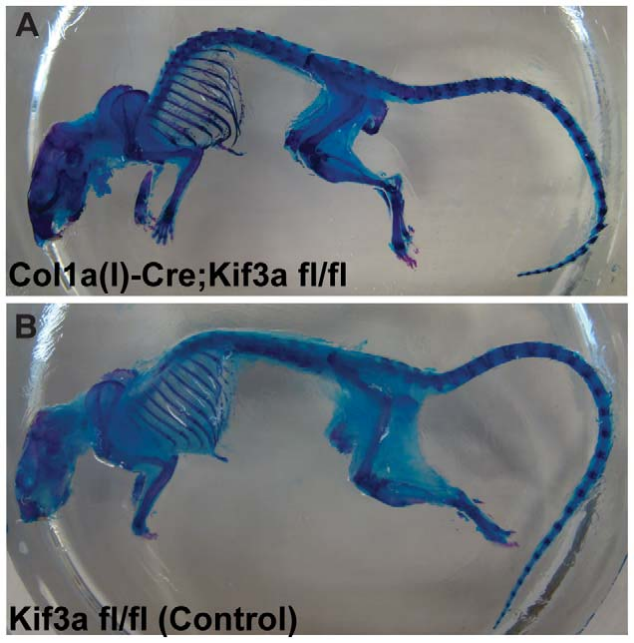
Skeletal morphology of adult *Colα1(I) 2.3-Cre;Kif3a^fl/fl^* mice is similar to control mice. (**A,B**) Comparison of 16 week old *Colα1(I) 2.3-Cre;Kif3a^fl/fl^* (A) and control (B) mice stained with Alizarin Red (bone) and Alcian Blue (cartilage) revealed no differences in size or morphology of the axial or appendicular skeleton.

We then assessed whether mature *Colα1(I) 2.3-Cre;Kif3a^fl/fl^* mice exhibit altered skeletal morphology. *Colα1(I) 2.3-Cre;Kif3a^fl/fl^* and control mice were compared using whole mount staining and micro-computed tomography. There were no significant differences in the size of adult skeletons between *Colα1(I) 2.3-Cre;Kif3a^fl/fl^* and control mice (Fig. 3). There were also no significant effects of *Kif3a* deletion on ulna length or cortical bone architecture at the ulnar midshaft (Table 1). Although most of the static histomorphometric measurements of trabecular bone did not show a difference between control and cKO animals (Table 2), there were some minor differences. For example, there was a 4% higher bone volume fraction in the distal femur that was also reflected in altered trabecular number and spacing. In all cases the differences were statistically significant, but small in magnitude and often inconsistent. Thus, they appear to be statistical anomalies rather than evidence of altered bone architecture. Taken together, these data suggest that disruption of intraflagellar transport in osteoblasts and osteocytes did not affect bone formation during development, and resulted in no difference in skeletal morphology between adult *Colα1(I) 2.3-Cre;Kif3a^fl/fl^* mice and controls.

**Table 1:** *Kif3a* deletion in osteoblasts and osteocytes has no effect on tibial midshaft geometry.

	Control (n = 25)	Col2.3-Cre;Kif3a fl/fl (n = 22)	p-value
***Ulna***			
Bone Length (mm)	14.4 ± 0.2	13.99 ± 0.2	N.S.
Imin (mm^4^)	0.0046 ± 0.0002	0.0048 ± 0.0002	N.S.
Imax (mm^4^)	0.0160 ± 0.0009	0.0147 ± 0.0006	N.S.
***Tibia Midshaft***			
Cortical Thickness (mm)	0.238 ± 0.003	0.239 ± 0.008	N.S.
Imin (mm^4^)	0.077 ± 0.005	0.066 ± 0.006	N.S.
Imax (mm^4^)	0.128 ± 0.006	0.109 ± 0.010	N.S.
pMOI (mm^4^)	0.205 ± 0.011	0.175 ± 0.016	N.S.

Imin and Imax are maximum and minimum second moment of inertia, respectively. pMOI is polar moment of inertia. Cortical bone geometry in 16 week old skeletally mature *Colα1(I) 2.3-Cre;Kif3a^fl/fl^* and control mice was assessed using as microCT. Data presented as mean±SEM. N.S. is not significant (p>0.15).

**Table 2:** *Kif3a* deletion in osteoblasts and osteocytes has minimal effect on trabecular bone architecture.

	Control (n = 25)	Col2.3-Cre;Kif3a fl/fl (n = 22)	p-value
***Proximal Tibia***			
BV/TV (%)	12.6 ± 0.9	14.6 ± 1.3	N.S.
Tb.N (1/mm)	3.75 ± 0.21	4.41 ± 0.26	N.S.
Tb.Th (mm)	0.058 ± 0.002	0.055 ± 0.002	N.S.
Tb.Sp (mm)	0.289 ± 0.019	0.246 ± 0.02	N.S.
Conn.D (1/mm^3^)	60.4 ± 10.3	97.0 ± 16.5	N.S.
***Distal Femur***			
BV/TV (%)	15.1 ± 1.0	19.0 ± 1.5	0.036
Tb.N (1/mm)	4.31 ± 0.17	4.94 ± 0.18	0.019
Tb.Th (mm)	0.057 ± 0.002	0.055 ± 0.001	N.S.
Tb.Sp (mm)	0.238 ± 0.011	0.200 ± 0.009	0.014
Conn.D (1/mm^3^)	104.4 ± 12.7	141.1 ± 13.4	N.S.
***L5***			
BV/TV (%)	26.0 ± 0.9	24.1 ± 1.0	N.S.
Tb.N (1/mm)	4.35 ± 0.16	4.34 ± 0.16	N.S.
Tb.Th (mm)	0.64 ± 0.002	0.059 ± 0.002	0.043
Tb.Sp (mm)	0.236 ± 0.009	0.233 ± 0.009	N.S.
Conn.D (1/mm^3^)	119.7 ± 10.5	125.2 ± 8.5	N.S.

Trabecular bone volume fraction (BV/TV, %), trabecular number (Tb.N, µm^−1^), trabecular thickness (Tb.Th, µm), trabecular spacing (Tb.Sp, µm), and connectivity density (Conn.D, µm^−3^) of the proximal tibia, distal femur, and L5 vertebra were measured using microCT. Data presented as mean±SEM. N.S. is not significant (p>0.15).

**Figure 4:**
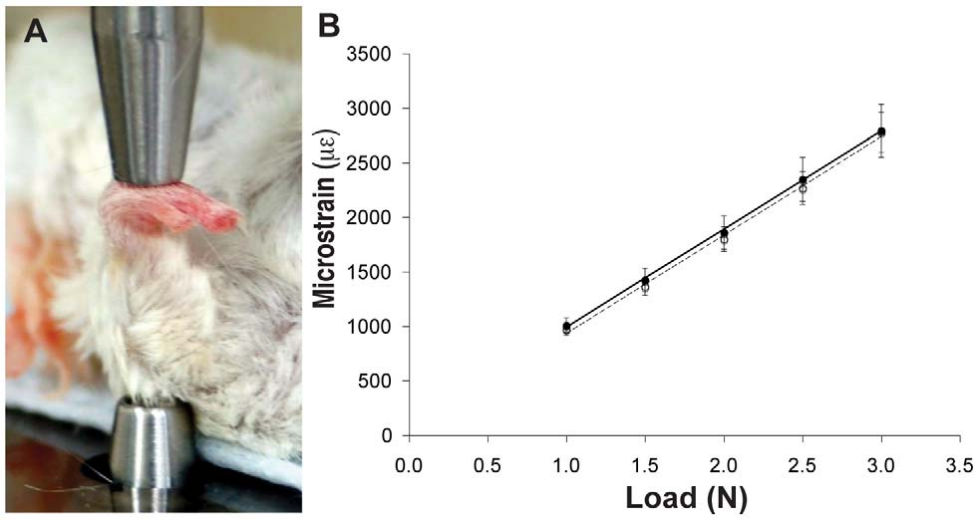
Axial ulnar loading leads to similar strain at the ulnar midshaft of *Colα1(I) 2.3-Cre;Kif3a^fl/fl^* and control mice. (**A**) Image of strain gaging and axial ulnar loading experimental set-up. The right forearms of 16 week old skeletal mature mice were axially loaded for 120 cycles per day for 3 consecutive days with a 2 Hz sine wave using an electromagnetic loading system with feedback control. The left forearms were not loaded and used as non-loaded internal controls. (**B**) Strain in cortical bone at given mechanical loading levels. Open and closed circles indicate *Colα1(I) 2.3-Cre;Kif3a^fl/fl^* (n = 35) and control (n = 27) mice, respectively. Data presented as mean ± SEM. * p<0.05.

Since the *Colα1(I) 2.3-Cre;Kif3a^fl/fl^* mice were devoid of developmental abnormalities observed with other *Kif3a* knockouts [24], [25], [26], we could determine whether primary cilia play a role in regulating mechanosensing in bone in the absence of complicating developmental effects. To ensure that we were introducing the same strain in both *Colα1(I) 2.3-Cre;Kif3a^fl/fl^* and control mice, we performed a strain calibration test prior to *in vivo* ulna loading studies (Fig. 4A). We determined that strain in cortical bone at a given load level was the same between *Colα1(I) 2.3-Cre;Kif3a^fl/fl^* and control mice (Fig. 4B).

**Figure 5:**
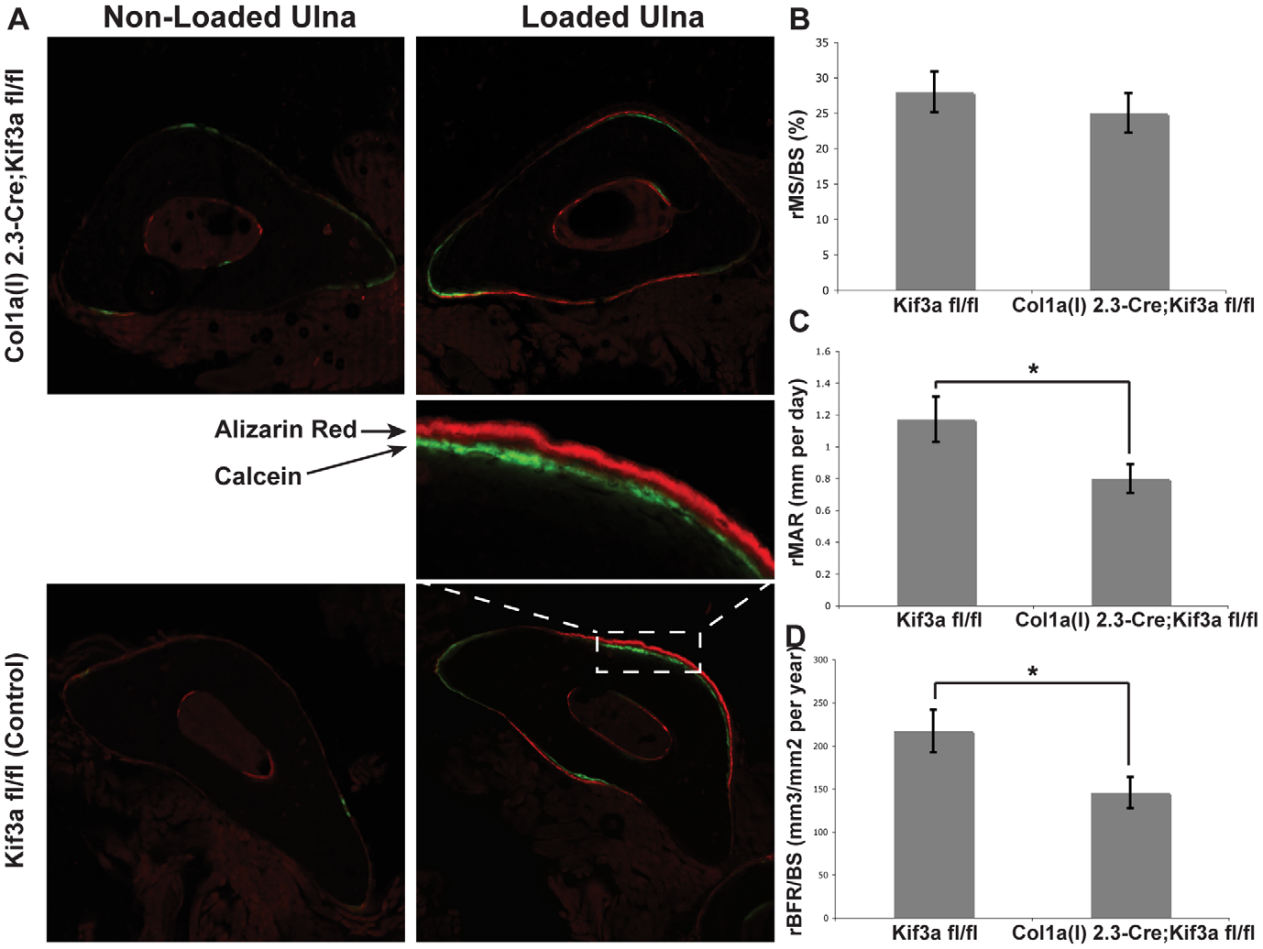
Skeletally mature *Colα1(I) 2.3-Cre;Kif3a^fl/fl^* mice exhibit less responsiveness to mechanical loading compared to control mice. (**A**) Representative images of non-loaded (left) and loaded (right) ulnae of *Colα1(I) 2.3-Cre;Kif3a^fl/fl^* (top) and control (bottom) mice. Fluorochrome labels (Calcein-green and Alizarin Red-red) given on Days 5 and 12 after the onset of mechanical loading. (**B to D**) Relative mineralizing surface (rMS/BS, %, B), mineral apposition rate (rMAR, µm per day, C), and bone formation rate (rBFR/BS, µm^3^/µm^2^ per year, D) of mechanically loaded mice. *Colα1(I) 2.3-Cre;Kif3a^fl/fl^* mice exhibited a decrease of 32% in rMAR and 33% in rBFR/BS when compared to control mice. Data presented as mean ± SEM. * p<0.05.

Mutants and littermate controls were then exposed to a cyclic ulnar loading regimen of one minute per day for three consecutive days (Fig. 4A) and bone formation was assayed with injected fluorescent tracers and dynamic histomorphometry (Fig. 5A) [27]. After 3 consecutive days of loading, there was a significant increase in rMS/BS (Fig. 5B), rMAR (Fig. 5C), and rBFR/BS (Fig. 5D) in both *Colα1(I) 2.3-Cre;Kif3a* mutant and control mice (Table 3). However, the increase was significantly less in *Colα1(I) 2.3-Cre;Kif3a^fl/fl^* mice, by approximately 32% in rMAR and 33% in rBFR/BS, as compared to control mice (Fig. 5C,D). Collectively, our data show that an osteoblast- and osteocyte- specific knockout of Kif3a avoids the developmental abnormalities of other primary cilium knockouts, but that primary cilia play a critical role in the response of adult bone to mechanical loading *in vivo*.

**Table 3:** *Colα1(I) 2.3-Cre;Kif3a^fl/fl^* were significantly less responsive to mechanical loading than control mice.

	MS/BS (%)		MAR (um per day)		BFR/BS (um3/um2 per year)	
***Col2.3-Cre;Kif3a fl/fl*** ** (n = 35)**						
left (non-loaded)	12.30+/−2.05		0.316+/−0.046		21.62+/−5.41	
right (loaded)	36.82+/−2.58	+++	1.089+/−0.086	+++	166.78+/−18.55	+++
relative (loaded - non-loaded)	25.01+/−2.79		0.798+/−0.090	*	145.52+/−18.23	*
***Control*** ** (n = 27)**						
left (non-loaded)	12.96+/−2.49		0.481+/−0.074		31.64+/−7.78	
right (loaded)	40.59+/−2.51	+++	1.644+/−0.120	+++	248.71+/−24.92	+++
*relative (loaded - non-loaded)*	27.98+/−2.88		1.171+/−0.143		217.07+/−24.62	

*Colα1(I) 2.3-Cre;Kif3a^fl/fl^* and control mice responded to mechanical loading with increased mineralizing surface (MS/BS), mineral apposition rate (MAR), and bone formation rate (BFR/BS), however, *Colα1(I) 2.3-Cre;Kif3a^fl/fl^* were significantly less responsive to mechanical loading than control mice. Data presented as mean±SEM.

+++ p<0.001 for loaded vs. non-loaded values.

* p<0.05 for *Colα1(I) 2.3-Cre;Kif3a^fl/fl^* vs. control mice.

## Discussion

The current primary cilium-related diseases are relatively rare, genetic disorders (e.g. Bardet-Biedl syndrome, Meckel-Gruber syndrome, and polycystic kidney disease), however, it is possible that primary cilia play a role in complex, multi-factorial diseases as well. For example, osteoporosis, a common and costly disease that fundamentally results from an imbalance of bone formation and bone resorption, can result from low levels of sex hormones, vitamin and mineral deficiencies, and lack of physical loading [28], [29]. In this study, we found that loading of *Colα1(I) 2.3-Cre;Kif3a^fl/fl^* mutant ulnae resulted in reduced loading-induced bone formation when compared to controls. We have previously shown that cultured osteoblasts and osteocytes respond to mechanical stimulation with primary cilia-dependent osteogenic gene expression [15]. Taken together these data support the idea that primary cilia may be more important in formation of bone in adults and in complex multi-factorial bone diseases, such as osteoporosis, than was previously appreciated.

In addition to their established role in development, our study provides further evidence that primary cilia play a role in maintaining adult tissue, specifically adult bone, due to mechanical loading. Recently, mice bearing global mutations in Pkd1 have been shown to form less cortical and trabecular bone with lower bone density and reduced osteogenic gene expression during skeletal development [16]. Further evidence suggests that Pkd1 might mediate sensing of mechanical stresses that occur with skeletal development [18]. In our study, there was no skeletal defect resulting from disrupting IFT in osteocytes and osteoblasts, suggesting that bone primary cilia do not participate in skeletogenesis. However, our loading results demonstrate that these primary cilia play an important role in mechanosensing in adult bone. There is precedence for the mechanisms of skeletal development and skeletal remodeling being distinct in the osteopontin-dependent response to mechanical loading [30] and integrin-dependent responses to mechanical loading and unloading [31], [32]. Thus, although the mechanisms of ciliary mechanotransduction in adult bone are not fully understood, our findings suggest that the primary cilium plays a significant role in the connection between extracellular sensing and bone formation in adult bone.

While numerous *in vivo* studies have shown that primary cilia act as chemosensors in several important chemical signaling pathways, there has been less *in vivo* evidence that primary cilia are involved in mechanosensing. Studies of primary cilia and mechanosensing have shown that there is a primary cilium-dependent Ca^2+^ response when cultured kidney cells are mechanically stimulated [8], [33]. More recently, *ex vivo* studies of primary cilia in the embryonic node and liver ducts implicated primary cilia as sensors of fluid flow [11], [14], [34]. In addition, Xiao et al. examined bone morphology of mice heterozygous for mutation of *pkd1*, a gene that encodes polycystin 1 [35]; polycystin 1 is a transmembrane protein that is found at the cilium, and the polycystin 1/2 complex is thought to have mechanosensing capabilities [36]. They found that these mice are osteopenic; however, mechanical loads were not applied directly [35]. Our results provide the first direct *in vivo* evidence that primary cilia in bone sense physical extracellular signals. They extend prior studies by demonstrating that primary cilia in bone sense physical extracellular signals and are important in cellular mechanosensing in bone in adults via a mechanism that might be distinct from the role of Pkd1 in skeletal development.

Because disrupting intraflagellar transport via the deletion of *Kif3a* may or may not result in missing or stunted primary cilia, demonstration of ciliary dysfunction is challenging. In vivo imaging of primary cilia would be inconclusive, and the associated technical difficulties have greatly limited the assessment of primary cilia in bone. Culturing primary osteocytes is another strategy, but the isolation of these cells and their return to a proliferative state would again make presence or absence of primary cilia, per se, inconclusive. Thus, verification of the Kif3a-deleted genotype, as shown in this study, may be the more effective approach.

This work raises a number of intriguing points for future research. There are a number of studies showing that disruption of IFT has led to a clear breakdown in signaling pathways, such as the hedgehog signaling [4], [21], [37] and Wnt signaling [5], [38]. In this study, we showed that response to loading was decreased in *Colα1(I) 2.3-Cre; Kif3a^fl/fl^* mice, and indeed, a number of studies have explored primary cilium-dependent mechanotransduction pathways in musculoskeletal systems [15], [39], [40]. However, we did not link disruption of any specific pathway to the deletion of Kif3a or primary cilia disruption in this study. In addition, while Kif3a is widely accepted to be essential to IFT in the primary cilium [19], [20] and Kif3a deletion to be a method of disrupting cilia formation and function [22], [41], [42], there are studies that show that Kif3a is associated with non-ciliary microtubules in some cell types [43], [44], [45]. Finally, there are a number of potential mechanosensors in bone, including integrins [31], [32], [46] and mechanically-activated membrane channels [47], [48], [49]. The existence of other mechanosensors is suggested by our studies since deletion of the Kif3a resulted in a decrease in loading-induced response when compared to wild-type mice, rather than a failure to respond to loading.

In summary, these findings represent an *in vivo* demonstration of a link between the disruption of primary cilia function and decreased bone mechano-responsiveness, suggesting that the primary cilium acts as cellular mechanosensors. Our results also suggest that primary cilia may play a broader role in regulating bone formation in adults and in complex multi-factorial bone diseases than was previously appreciated.

## Materials and Methods

### Animals

*Kif3a^fl/fl^* and *Colα1(I) 2.3-Cre* mice were created as described previously [22], [23]. Male *Colα1(I) 2.3-Cre* and female *Kif3a^fl/fl^* mice founder mice were used to generate *Colα1(I) 2.3-Cre;Kif3a^fl/fl^* (experimental) and *Kif3a^fl/fl^* (control) for experimentation. The *Cre* transgene was transmitted through the male to avoid undesirable Cre activity in the female germline. To assess Cre expression, *Colα1(I) 2.3-Cre* mice were mated with *Rosa26R* reporter mice [50]. For staged embryos, *Colα1(I) 2.3-Cre;Kif3a^fl/fl^* and *Kif3a^fl/fl^* mating pairs were set up in the afternoon and checked for vaginal plugs the next morning. Noon of the day of the vaginal plug was designated as embryonic day E0.5. Genotypes of transgenic mice were determined by PCR analysis of genomic DNA from tail biopsies. The heterogeneous mix of tail tissue, some of which did express *Cre* recombinase (e.g. bone) and some of which did not (e.g. skin, muscle, etc.), often resulted in a combination of floxed and recombined alleles in PCR results, depending on the portion of the tail biopsy that was digested. In these samples, a band representing the recombined allele was present at 200 bp. *Cre* and *Kif3a* floxed and wildtype primers were previously described [22], [23]. 18S was used as a positive control for the *Cre* PCR reaction with P1: 5′ CAA GGA AGG CAG CAG GCG CGC AAA T 3′; P2: 5′TGC ACC ACC ACC CAC GGA ATC GAG AA 3′. Mice were housed up to 5 per cage and provided with standard mouse chow and water ad libitum throughout the study. All procedures performed in this study were approved by the Palo Alto IACUC with ACORP protocol approval number JAC050806MOU.

### LacZ Staining, Histology and Whole Mount Skeletal Staining

Tissues were harvested at specified time points, fixed in 4% paraformaldehyde for 24 h at 4°C, decalcified for 10–14 days in 19% EDTA at 4°C, and embedded in paraffin or OCT. Sections were cut at 8–12 µm thickness. For histology, sections were stained with Movat's pentachrome [51]. Adjacent sections were stained with Picrosirius red and Safranin O. For LacZ staining, tissue sections were fixed, permeabilized, and stained for 4–5 days in a 1 mg/mL X-gal (5-bromo-4-chloro-3-indolyl-D-β-galactosidase) solution, adapted from Colnot et al. [52]. Whole mount skeletal staining with Alizarin Red and Alcian Blue was performed as previously described [53], [54].

### Micro-Computed Tomography

Bone samples from sixteen week old *Colα1(I) 2.3-Cre;Kif3a^fl/fl^* and control mice were dissected, fixed in 70% ethanol, and scanned using micro-computed tomography (Scanco vivaCT 40, Bassersdorf, Switzerland) at 10 µm isotropic resolution. Cortical bone analysis on the mid-diaphysis included cortical thickness and maximum and minimum second moment of inertia (Imax and Imin). Trabecular bone analysis was completed at the proximal tibia, distal femur and L5 lumbar vertebra. Trabecular bone was designated using contours inside the cortical shell on two-dimensional slices. Histomorphometric analysis included bone volume fraction (BV/TV, %), trabecular number (Tb.N, µm^−1^), trabecular thickness (Tb.Th, µm), trabecular spacing (Tb.Sp, µm), and connectivity density (Conn.D, µm^−3^).

### Strain Measurement During Axial Ulnar Loading

Strain calibration test were performed prior to the *in vivo* ulna loading study to ensure that the same strain was introduced in both *Colα1(I) 2.3-Cre;Kif3a^fl/fl^* and control mice. Peak mechanical strain at the medial aspect of the ulnar midshaft was measured based on a previously described protocol [55]. The forearm of 16 week old *Colα1(I) 2.3-Cre;Kif3a^fl/fl^* and control mice were loaded in axial compression, using the same device and frequency used for *in vivo* loading (Fig. 3A). Forearms were loaded at magnitudes of 1.0, 1.5, 2.0, 2.5, and 3.0 N, during which peak-to-peak voltage was measured using a digital oscilloscope. Voltage was converted to strain as previously described [55]. We determined that strain in cortical bone at a given load level was the same between *Colα1(I) 2.3-Cre;Kif3a^fl/fl^* and control mice (Fig. 3B).

### In Vivo Axial Ulnar Loading

The *in vivo* axial ulnar loading protocol was modified from Robling and Turner [55]. Because the experimental animals were of mixed genetic background resulting from the cross of C57Bl/6 and FVB mice, we did not know a priori what load level would produce a robust bone formation. Thus, we conducted an initial dose-response experiment with 2.8N, 3.0N, and 3.4N applied load. A consistent formative response was found in the 3.4N group only, thus we chose this load level for the in vivo axial ulnar loading experiments. While under isofluorane anesthesia (2.5%) the right forearm from each mouse was axially loaded for 120 cycles per day for 3 consecutive days with a 2 Hz sine wave using an electromagnetic loading system with feedback control (Fig. 3A; EnduraTEC, Bose, Eden Prairie, MN). The left forearms were not loaded and used as non-loaded internal controls. All mice were allowed normal cage activity in between loading sessions. All mice received subcutaneous injections of Calcein (30 mg/kg body weight; Sigma Chemical Co., St. Louis, MO) 5 days after the first day of loading and Alizarin Red (50 mg/kg body weight; Sigma Chemical Co., St. Louis, Mo, USA) 12 days after the first day of loading. All animals were euthanized 19 days after the first day of loading and processed for dynamic histomorphometric analysis

### Dynamic Histomorphometry

The right and left ulnae were isolated, cleaned of soft tissue, measured for total length and stored in 70% ethanol for histomorphometry. The ulnae were then dehydrated in graded alcohol (70–100%), infiltrated with three changes of methyl methacrylate (Aldrich Chemical Co., Milwaukee, WI), and embedded in methyl methacrylate in the presence of benzoyl peroxide. Transverse sections of the ulnar midshaft were collected using a diamond saw (Isomet, Buehler, Lake Bluff, IL) and imaged on a laser scanning confocal microscope (Nikon C-1) using a 0.45 NA 10× objective.

Using Image J, the following data were collected from the periosteal surface: bone perimeter (B.Pm), single label perimeter (sL.Pm), double label perimeter (dL.Pm), and double label area (dL.Ar). From these primary data, the following were calculated for each bone: mineralizing surface (MS/BS = [1/2 sL.Pm+dL.Pm]/B.Pm×100; %), mineral apposition rate (MAR = dL.Ar/dL.Pm/7days; µm per day) and bone formation rate (BFR/BS = MAR×MS/BS×3.65; µm^3^/µm^2^ per year). Relative (r) measurements of rMS/BS, rMAR, rBFR/BS, were calculated by subtracting the non-loaded from loaded values to show increases due to mechanical loading.

### Statistical Analysis

Data were expressed as mean ± SEM. Differences in slope and intercept for the linear regressions between *Colα1(I) 2.3-Cre;Kif3a^fl/fl^* and control mice were analyzed by analysis of covariance. Differences between right (loaded) and left (non-loaded) ulnae were analyzed using paired t-tests (two-tailed). All other comparisons between *Colα1(I) 2.3-Cre;Kif3a^fl/fl^* and control mice were analyzed using unpaired t-tests (two-tailed). For all tests, α = 0.05.
